# The Role of Online Social Support in Supporting and Educating Parents of Young Children With Special Health Care Needs in the United States: A Scoping Review

**DOI:** 10.2196/jmir.6722

**Published:** 2016-12-22

**Authors:** Beth A DeHoff, Lisa K Staten, Rylin Christine Rodgers, Scott C Denne

**Affiliations:** ^1^ Neonatology Department of Pediatrics Indiana University Health Physicians Indianapolis, IN United States; ^2^ Department of Social and Behavioral Sciences Richard M. Fairbanks School of Public Health Indiana University Indianapolis, IN United States; ^3^ Riley Child Development Center Department of Pediatrics Indiana University School of Medicine Indianapolis, IN United States; ^4^ Neonatology Department of Pediatrics Indiana University School of Medicine Indianapolis, IN United States

**Keywords:** health communication, child, social media, health education, health resources, early childhood, disability, neonatal intensive care unit, family, maternal-child health services

## Abstract

**Background:**

When parents of young children with special health care needs (CSHCN) receive their child’s diagnosis, they encounter information they may not understand, emotions they may not know how to cope with, and questions about their child’s immediate and long-term future that frequently lack answers. The challenge of health care providers is how to prepare parents for caring for their CSHCN, for coping with any resulting challenges, and for accessing the systems and services that can assist them.

**Objective:**

The purpose of this work was to review evidence of the information and support needs of parents of young CSHCN and to determine whether online social support can serve as an avenue for learning and empowerment for these parents.

**Methods:**

A scoping review identified the challenges, coping mechanisms, and support needs among parents of CSHCN, and the reach and effectiveness of digital technologies with these families and health care providers. We also conducted interviews with professionals serving parents of CSHCN.

**Results:**

The literature review and interviews suggested that parents best learn the information they need, and cope with the emotional challenges of raising a CSHCN, with support from other parents of CSHCN, and that young parents in recent years have most often been finding this parent-to-parent support through digital media, particularly social media, consistent with the theory of online social support. Evidence also shows that social media, particularly Facebook, is used by nearly all women aged 18-29 years across racial and socioeconomic lines in the United States.

**Conclusions:**

Parents of young CSHCN experience significant stress but gain understanding, receive support, and develop the ability to care for and be advocates for their child through parent-to-parent emotional and informational social support. Online social support is most effective with young adults of childbearing age, with social media and apps being the most useful within the theoretical framework of social support. This opens new opportunities to effectively educate and support parents of young CSHCN. Providers seeking to inform, educate, and support families of CSHCN should develop strategies to help parents find and use social support through digital resources to facilitate their emotional adjustment and practical abilities to care for and access services for their child.

## Introduction

Parents of infants and young children with special health care needs (CSHCN) are often thrust into a world they know little about—a vast network of professionals, systems, and services that address the needs of children with disabilities. (In this paper, “parents” refers to guardians of all types.) At the same time, these young parents must learn to cope emotionally and practically with a child’s medical and developmental needs, a situation far from the dreams of many young parents. These challenges can confront young parents when their child receives a diagnosis as a young child delayed in reaching milestones, as a newborn requiring a neonatal intensive care unit (NICU) stay for special health care needs, or even in pregnancy with a prenatal diagnosis. A key challenge for professionals who serve these families is in helping them to gain emotional support and, subsequently, the informational knowledge and skills necessary to be advocates for and achieve better health outcomes for their children. Improving social support is one way to accomplish this. Increasingly, digital communication, and social media in particular, are being used to offer online social support for parents of CSHCN. Given that research on digital communication with parents of CSHCN is lacking, we address the following question: Can we expect that social media can be an effective avenue for emotional and informational support for parents of CSHCN? In attempting to answer this question, we offer a novel exploration of how established benefits of parent-to-parent support and the theoretical framework of online social support can provide new avenues for professional partnerships to support families of CSHCN.

### Challenges to Obtaining Necessary Support for Parents of CSHCN

CSHCN are defined by the US Maternal and Child Health Bureau as “those who have or are at increased risk for a chronic physical, developmental, behavioral or emotional condition and who also require health and related services of a type or amount beyond that required of children generally” [1]. When parents give birth to a CSHCN, or receive such a diagnosis prenatally or in the early years of childhood, they are immediately faced with a loss of the child they thought they would have and a tremendous amount to learn. The result is emotional stress that is overwhelming as parents sort through medical and developmental information, resources and services, varying reactions from friends and family, and even adjusting to their own new identities as parents of a CSHCN [2,3].

Shortly after a new diagnosis of a child’s special health care need, parents typically receive education from their child’s hospital NICU or specialist—often at one in-person appointment—about caring for their child and referrals for medical and community-level assistance. This usually occurs in the hospital just before discharge from the NICU, or in a pediatric specialty office at the end of a diagnosis appointment. At these early stages, however, parents are typically passive receivers of information as they try to process difficult information without the emotional capacity or skill sets developed to ask questions of care providers, or even to know what to ask. Mishel’s theory of uncertainty in illness proposes that stressful health care experiences such as new diagnoses actually interfere with the ability of patients and families to process and understand information shared with them [4]. Only later do they start to comprehend their situation and begin to seek additional support, sometimes from care providers, but more often from family, friends, and the Internet [5]. As parents adjust to the diagnosis and start to try to make sense of how to proceed with their lives and help their child, they report that their child’s medical and even social work providers have inadequate and sometimes inaccurate information about services to help the child and family at home and in their community. Initially, many parents rely on existing support networks such as family or friends who have always been their main source of all kinds of support. In many cases, these individuals lack an understanding of the child’s diagnosis and resulting challenges. For emotional understanding and resources, parents have indicated that they prefer to connect with other parents of CSHCN who live in their geographic area and who have a child with the same or a similar diagnosis [6,7].

### Parent-to-Parent Support as an Avenue of Social Support for Parents of CSHCN

The concepts of social support and parent-to-parent support for CSHCN both had their start in the 1970s. In 1976, Cassel concluded from both human and animal studies that social support mediates the health impact of stress [8]. In 1981, House identified four kinds of supportive actions that can help people deal with a crisis or chronic stress and allow them to cope, learn, and even grow during their challenges. This support can be emotional (being there), instrumental (doing things), informational (sharing knowledge and resources), or appraisal (helping individuals to see their stressors with more confidence in their ability to cope). Such support can provide a person with the strength to find new contacts and information in order to solve problems, the cognitive skills necessary to process that information, and the ability to better cope with stress, doubt, and fear [8]. For many young parents of CSHCN, empowerment gained from social support—particularly a combination of emotional and informational support—can lead them to successfully navigate the medical information and services necessary to help their child.

**Benefits of parent-to-parent support (adapted from Santelli et al [9]).**

- Increased acceptance of child’s special health care needs
- Enhanced parent coping skills
- Increased self-efficacy for parents to work on problems and access services
- Rated as helpful by more than 80% of parents served
- Access to support typically unavailable from any other source
- An essential aspect of family-centered care

The idea of parent-to-parent support for families of CSHCN grew out of the movement for family-centered medical care beginning in 1971. Since that time, veteran parents of CSHCN supporting new parents has been promoted as a way for families to both reduce stress by realizing they are not alone in their struggles, and as a way to find solutions and services for their CSHCN. At first, parent-to-parent support was facilitated by health care professionals who connected a parent to another parent whose child had a similar diagnosis. Over time, organizations devoted to providing support to families of CSHCN through other parents emerged. Research into parent-to-parent support in the 1990s showed that this type of social support has several benefits, as Textbox 1 shows [9].

Parents of children with a variety of special health care needs who have been mentored by other parents of CSHCN have reported increased emotional well-being and better adaptation to their new life and identity as the parents of a CSHCN. A 2007 study of parents of CSHCN found benefits of parent-to-parent support as well, reporting that relationships with other parents helped them share experiences, feel less alone, and even find positive aspects of very painful experiences [10]. While this study focused on personal and phone support, Konrad noted that the Internet and email listservs offered additional avenues for parent-to-parent support that bridge barriers of geographic distance and rare disorders. An email support forum for parents of children with clubfoot was shown to help families, especially mothers, gain information and manage uncertainty about their child’s diagnosis [11]. Today, listservs and email are fading in popularity, while social media and mobile phone apps are becoming more popular [12]. A study of military families found a preference for online parent support via Facebook over face-to-face support, particularly among mothers [13]. Regardless of format, the reported benefits of parent-to-parent support remain consistent across time and modalities.

Traditionally, health education and social support for parents of CSHCN were available through in-person trainings and support groups. Leaders of family support nonprofit organizations for families of CSHCN responded to a common interview protocol in ways that reinforce the findings of the literature. “Support used to happen in group trainings and over kitchen tables,” said Jane Scott, assistant director of About Special Kids in Indiana, USA, a not-for-profit organization that provides parent-to-parent support for Indiana parents of CSHCN. “Now it’s social media. It’s not the same as in-person support, but it has reduced families’ isolation, and it’s especially great for rural families without options for in-person groups nearby.” Scott also noted that short message service (SMS) text messaging with families seeking information has been highly effective, with 80% of parents responding to a text message, more than twice that of parents who return a call (J Scott, oral communication, January 2016). Likewise, Jennifer Akers, project director of Family Voices Indiana, noted that parents like in-person training but, because they often lack time to attend, many interact on Facebook and “tag” her with questions. Family Voices Indiana is an Indiana nonprofit focused on parent-to-parent support for CSHCN, particularly in the areas of health care financing and advocacy. Akers said the importance of parent-to-parent organizations is that they can explain things in ways that parents understand and can apply to their lives, which she says often works better for families than information they receive from clinical and social services professionals (J Akers, written communication, January 2016). A 2014 study noted that low-income families have a particularly difficult time attending in-person support and education programs, with a recruitment rate of 31% and a high rate of attrition due to problems with child care, transportation, and time and work constraints. Swindle and colleagues found widespread access to the Internet, mobile phones, and social media among the same low-income parents, suggesting that, for low-income parents, technology may be a more accessible path to education and support than in-person programs [14]. The experiences of these nonprofit organizations serving families of CSHCN support those findings.

### Theoretical Frameworks: Theory of Social Support

The availability of social supports helps individuals believe they are well supported, which leads them to interpret others as supportive, have a better ability to draw from past support, and have the ability to readily think about their current sources of support. This social cognitive perspective of social support [15] proposes that perceived support—an individual’s belief that she is well supported—leads to better coping skills and higher self-esteem. Individuals under stress engage in appraisals to determine whether a stressful situation is a genuine threat, and whether they have the personal and social resources to deal with the situation.

**Figure 1:**
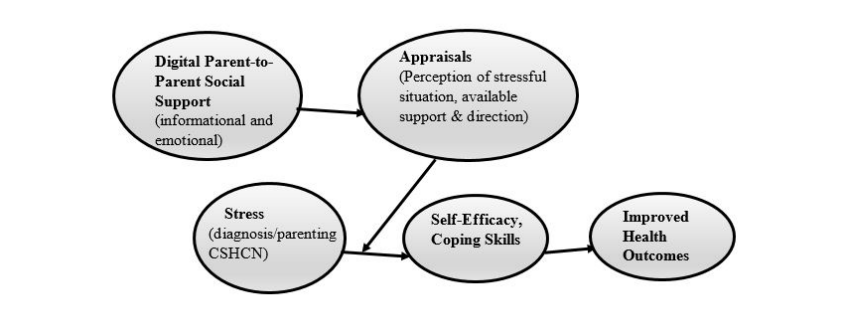
Digital parent-to-parent social support as a path to better outcomes for children with special health care needs (CSHCN). Adaptated from Lakey and Cohen’s social support theory [15].

Parents of newly diagnosed young CSHCN face the stressful situation of receiving a diagnosis of their child’s condition that brings significant uncertainty and fear. However, the skills and self-confidence built through social support can empower parents to interact more positively with care providers and medical information [16]. The model shown in Figure 1 adapts Lakey and Cohen’s [15] model of the social support theory to show how emotional and informational support available from contact with other parents of CSHCN affects how parents perceive the stressful situation with their child and the supports they can count on, which mitigates stress and leads to better self-efficacy and coping skills. This belief in social support and the resulting self-efficacy ultimately allow parents to understand their child’s care, seek important services for their children, and thus gain the potential to experience better health outcomes for both parent and child.

Social support can come from existing social networks (such as friends and family), new social networks (such as with other families sharing a similar diagnosis or experience), or from indigenous or community health workers. While many social service professionals focus on helping individuals strengthen their natural supports through family and friends, interventions for parents of young CSHCN also should focus on developing new social networks that exist around parenting CSHCN or dealing with specific diagnoses. New social networks often work best in response to a major life change or a specific stressor, such as a child’s diagnosis. People with similar experiences can provide support based on their like journeys, providing emotional and informational support from a viewpoint only someone with similar experiences can provide [8]. Caregivers of children with life-threatening or -altering conditions share an experience unlike any that most of their friends and families can understand. Without connections to other, similar families, parents of young CSHCN often feel isolated, overwhelmed, uninformed, and uncertain. Many identify a desire to access other families with the same diagnosis for advice and support [16].

### Informational and Emotional Support of Parents of CSHCN

Parents of children newly diagnosed with diabetes reported feeling incompetent, stressed, and anxious, and having low confidence when faced with learning how to administer home insulin injections, blood tests, and other care in a short period of time [17]. Parents indicated that the large amount of information was overwhelming and asked for repeated, short teaching sessions. In addition, parents indicated they wanted realistic, truthful information that includes not just instructions about how to care for their child but reasons why this care is needed, in ways that are practical for use in their lives at home [18]. Among parents of premature babies, parents are typically unable to actively engage in learning about their child’s special health care needs immediately after diagnosis [5]. Once parents become ready to seek information, as indicated by a survey of parents of young CSHCN, their top needs for information include finding ways to help their child develop, handling the emotional impact of having a CSHCN, managing the demands on the parents’ time, finding and accessing community resources, understanding their child’s rights, and planning for the future [19]—needs that reflect the inextricable connection between emotional and informational social support. These needs also reflect practical, real-life coping questions that may best be answered by other parents who have learned to navigate similar needs in their own children. The 2009-2010 US National Survey of Children with Special Health Care Needs showed similar needs among parents, while also identifying additional stressors around paying for needed services, especially in the common situation of one parent leaving employment in order to care for the child [12]. Several studies have suggested that stress can be alleviated through social support, and some have concluded that learning is difficult until the stress of a situation is reduced [8,19].

The desire for social support as a means to learn about and access services is consistent with the instrumental aspect of the social support theoretical framework [15]. Given the lack of support available in one’s own circle of family and friends, a lack of information in small or rural communities, and the previously referenced barriers to attending in-person training and support groups [14,20], this support is difficult to access for many families. As options for online support have grown, many parents of CSHCN are turning to online social support for information on systems and services that could benefit their child [16].

### Online Social Support of Parents of CSHCN

The online social support theory [21] expands the theoretical framework of social support to include sources of support found on the Internet. This theory is based on the assertion that a change or perceived change in health can bring on acute episodes of stress that, without relief of the condition, can lead to chronic stress. The ability of a person to adapt to significant changes, such as receiving a diagnosis of a special health care need in a young child, is influenced by individual and health factors, demographics, and, increasingly, Internet use. The online social support theory proposes that online support, particularly through social media, is especially helpful for caregivers of relatives with health concerns, including parents of CSHCN [22,23].

Healthy People 2020 emphasizes health communication and information technology as key methods to improve health outcomes and increase health care equity and quality, stating specifically the role of technology in widening social support networks [24]. Digital technologies such as mobile health apps, social media groups, and other Web-based resources are 21st century solutions that directly address the need for accessible health information and communication [25]. Online support and education have been increasingly used in health care and other interventions in a variety of contexts, including with families of children with diabetes, asthma, attention-deficit/hyperactivity disorder (ADHD), and hydrocephalus [2,26,27]. The ability of these digital technologies to provide social support to parents of CSHCN achieves the traditional aims of education and information sharing through a digital avenue of online emotional and informational support. The accessibility of these technologies to parents across racial and socioeconomic lines is expanding every year.

Social media sites, particularly Facebook groups for parents of CSHCN, have high levels of intimacy and immediacy, meaning that support is available despite members’ distance from one another, which naturally leads to high levels of social support. Social support is most helpful when it comes from others with similar experiences. Online peer support can provide instrumental support of information and resource sharing, as well as emotional support of caring, compassion, and inclusion [16]. Other forms of digital communication, such as blogs, listservs, chat rooms, and apps to record and share a child’s milestones or health care experiences, can also provide social support but generally work best as an adjunct to Facebook groups, which are the most interactive and interpersonal formats [23]. When used by parents of CSHCN, online support has been shown to reduce isolation, increase feelings of self-control, increase parents’ feelings of connection to others who understand, increase self-confidence, and lower depression and anxiety [27].

Numerous studies have concluded that mobile phone, Internet, and social media use crosses demographic lines such as income, race, and age [14,28-30]. Parents of young children have been using digital communication and social media since their teens or early adulthood, and they are already accustomed to connecting via apps, texting programs, and social media sites for social support [31]. All kinds of digital communication technologies—mobile phones, smartphones, Internet, mobile apps, Facebook, and other social media use—are most commonly used by the youngest group of adults: 18- to 29-year-olds. In this age group, the most common age group of parents of young children, shrinking social disparities in the use of these technologies are becoming even smaller. For instance, in the United States, the most prevalent users of the Internet and mobile phones are African Americans, and the most prevalent users of Facebook are Hispanics [14,28-30] (Table 1). Some studies, however, have found lower overall Internet use among minorities with low levels of education [32].

**Table 1:** Access to digital technologies for social support across US demographic groups^a^.

Demographic groups		Access
**Annual income (US $)**		
	High (75,000)	98% own mobile phones 78% use Facebook 84% own smartphones
	Low (<30,000)	84% own mobile phones 73% use Facebook 50% own smartphones
**Age range (years)**		
	18-29	90% use social media 86% own smartphones
	30-49	83% own smartphones
	All adults	90% own mobile phones 85% access Internet 64% own smartphones
**Racial/ethnic/geographic groups**		
	African Americans	68% own smartphones
	Hispanics	64% own smartphones
	Whites	66% own smartphones
	Rural Americans	75% view mobile health services as important

^a^Data from [14,28-30].

### Provider Use of Online Social Platforms

Hospitals and health care providers are increasingly communicating digitally with one another and, less often, with patients. A survey of outpatient family practice patients revealed that 56% said they wished their doctor used social media to interact with them. Despite this interest, many clinicians have been hesitant to enter into electronic health communication with patients due to US Health Insurance Portability and Accountability Act regulatory concerns over privacy and well-publicized, inadvertent breaches of patient confidentiality by health care professionals [33]. However, most privacy breaches have been around professionals’ posts on their personal social media pages, and not from official health care social media pages. In fact, health care providers are able to communicate via social media with a patient if the patient is the only one who can see it or if the patient has consented to communication via the digital platform [34]. The overriding principle in providers’ involvement in digital communication efforts in general, and in social media in particular, is that the patient must be in control of their participation. It is also possible that, just as young adults are the highest users of social media, there may be generational differences in physicians’ willingness to engage with patients and families online. Yet with widely available opportunities to connect online with peers beyond their own communities, and with few health care providers engaging with patients in this way, patients and families are increasingly relying less on medical providers and more on their peers for health information [35].

The findings about digital health communications in the literature are consistent with the experiences of Nerissa Bauer, MD, a pediatrician involved in research on parent engagement among children with ADHD at the Indiana University School of Medicine, Department of Pediatrics, Children’s Health Services Research. Bauer has developed support groups for parents and children with ADHD and reports that getting parents to attend is a “logistical nightmare” and meets with considerable parent resistance. However, she says that parents who attend enjoy the in-person group and want to continue. Even so, she has found that parents also want information online in general and through social media particularly, echoing results from much of the literature. “Parents say they want ways to interact with health information for their kids online, but we’ve found that if it’s not on Facebook, if it’s on a different platform than what they already use, they don’t use it,” she said. At the same time, she said, hospitals and academic centers are extremely cautious about using Facebook for reasons relating to privacy and the expectations of parent-professional interaction online, a situation also noted in the literature. “It’s definitely something to think about, and parents want it, but there are real hurdles to making it work,” she said.

Currently, Bauer is working on parent-developed YouTube videos of family members talking about life with their child or grandchild with ADHD, with the idea that parents who help develop the video will share the video on their own social media channels, a video approach to parent-to-parent social support. “A mom can watch these in her pajamas at 3 AM and find families who share her experiences,” Bauer said. This project is early in development and has not yet been implemented and evaluated. She also has found blogs have been a successful and controlled way to share information with parents one-way rather than interactively, but acknowledges that this strategy is better for education and information sharing than for social support (N Bauer, MD, oral communication, March 2016).

Academic literature and input from family and health leaders all point to the benefits of parent-to-parent communication and support, as well as barriers that parents of CSHCN face in finding the time and means to meet with other parents in similar situations. The aim of this scoping review was to consider what research to date has shown regarding the usefulness of digital communication, and in particular social media, in providing informational and emotional support to CSHCN.

## Methods

The observations and recommendations in this paper focus on the information needs of parents of CSHCN, the barriers to face-to-face education and communication for this population, and the effectiveness of electronic media for health education and communication with parents and others using eHealth sources. It is based on a scoping review conducted in PubMed, Social Sciences Citation Index, ACM Digital Library, Education Resources Information Center (ERIC) accessed through ProQuest, and Google Scholar. Scoping reviews, like summary reviews, use rigorous and transparent research methods but focus on the findings of the reviews rather than the research used to obtain those findings [36]. We searched the databases using the following phrases, individually and in combination: “children with special needs,” “children with special health care needs,” “children with disabilities,” “parents of children with special needs,” “parents of children with special health care needs,” “parents of children with disabilities,” “parents of young children with special needs,” “parents of young children with special health care needs,” “parents of young children with disabilities,” “digital communication,” “e-health,” “electronic communication,” “electronic health education,” “health communication,” “social media”, “apps,” and “texting.” The search also included results found in PubMed’s related articles feature. We eliminated articles focusing on digital health interventions for children rather than parents, and digital health interventions for adults not targeted as parents. We also eliminated articles on health education and communication methods published prior to 2005, with searches focused on digital health education and communication articles from 2010 or later. Articles focused on the needs and challenges of parents of CSHCN were selected due to their relevancy to the topic rather than their date of publication. In each article we used, the focus was on the information and communication needs of the audience, the methods of health communication and education used, and the results for different racial and socioeconomic groups.

The collection of data also looked specifically at the use of social media, mobile phones, and other technology, and barriers to accessing various forms of education and communication, particularly among parents of CSHCN. As the literature revealed the information preferences and barriers for parents of CSHCN, it became apparent that the theories of social support and online social support were important theoretical frameworks for the subject, resulting in subsequent searches of the databases for “social support,” “online social support,” and both of these terms with the term “theory” and “theoretical framework.” Searches for health care providers’ use of social media included the terms “hospital use of social media” and “physician use of social media,” and each of these terms with “with patients” added. This was not a systematic review of the efficacy of eHealth interventions, so although the literature we present covers every subject explored in the search, it includes the sources most illustrative of these related topics rather than every article we found in the literature.

We gleaned supporting information from key-informant conversations with leaders at four Indiana organizations that serve families of CSHCN. These conversations focused on how parents engage with the organization face-to-face and online, and what strategies for informing and supporting families of CSHCN have been most successful. Another key informant was an Indianapolis pediatrician engaged in services for and research regarding children with ADHD, who discussed her work with a parent support group and her thoughts about online support for these parents.

## Results

Parents of young CSHCN have enormous needs for informational and emotional support, and they often express the desire to connect with other parents for help with these needs. Juxtaposed with these needs for social support are barriers to attending in-person parent trainings or meetings, issues felt most acutely by caregivers of CSHCN who are isolated by geography or the intense care needs of their child. While research into digital communication for parents of CSHCN is limited, each study that has reported on the issue reveals that using online sources for information and support is not only possible for parents of CSHCN, it’s already happening outside of the health arena. Parents of CSHCN are already using social media, particularly Facebook, to seek out parent-to-parent online social support, and these parents report the online groups are helpful in providing informational and emotional support. These effects are consistent with the social cognitive theory of online social support [21] and social cognitive perspective of social support [15], which proposes that online support enhances participants’ belief that they are supported, affecting their perception of stressful situations and allowing them to develop coping skills that lead to better health outcomes.

In seeking effective ways to communicate with, educate, and support parents of young CSHCN, the theoretical framework of social support illustrates a way toward future efforts. For decades, parent-to-parent support has been a key pathway toward parents’ knowledge and understanding of services and supports for their CSHCN and how to access them, within the context of emotional support. This existing structure reflects the social support theory, that individuals with good social support in a crisis or time of significant change, particularly from individuals with similar experiences, are better able to cope with and move forward in the situation. Today, barriers to attending in-person programs and the availability of online social networking has moved parent-to-parent support to a largely digital venture, primarily through Facebook. This online support, consistent with the theory of online social support, can buoy parents with coping skills and self-efficacy, as well as information, to allow them to better care for and be advocates for their children.

The literature about how parents of CSHCN find support is echoed in the words of the physicians and professionals in personal interviews, and reinforced by other evidence found in randomized controlled trials about health communications. Many of these studies have concluded that texting, mobile apps, and social media are all effective ways of educating and supporting people facing serious life changes or health issues, particularly among younger adults such as parents of young children [31,37]. Following is a look at the literature involving each of these digital formats.

### Mobile Phones and Texting

Texting is perhaps the first digital technology used for health communication, and it still is in use today, with uses ranging from appointment reminders, to health behavior prompts, to health information messages [38]. The ability to send and receive text messages is widespread in the United States, with 90% of all adults owning a mobile phone. Among adults 18-29 years old, 98% have a mobile phone, as do 97% of adults age 30-49 years [39]. This popularity gave rise to the text4baby program, which debuted in 2010 as a way to deliver prenatal and postpartum health messages to pregnant women and mothers who recently delivered, with messages timed to the baby’s gestational age based on due date. As of February 2016, text4baby had served almost 975,000 unique pregnant and recently delivered women [40]. In 2014, a study of the use of text4baby by 943 women at an army medical center found that text messages affected the women’s beliefs about health issues, particularly the importance of taking prenatal vitamins, the importance of seeing a health care provider, and the risks of drinking alcohol during pregnancy. It did not, however, seem to affect health behaviors [41]. Other studies found that text health programs had significant effects on smoking cessation and mixed effects on diabetes management and asthma management, with limited evidence and a lack of research into the long-term effects of health text programs [27,42]. Researchers have noted that studies of texting as a health intervention in general is difficult due to the variety of strategies, conditions, and subjects involved, making it unrealistic to evaluate effectiveness of texting interventions outside of small programs focused on a narrow subject, such as diabetes [38].

The literature we searched revealed no studies related to texting interventions for parents of CSHCN, and no studies relating text messaging to social support. Texting in health care has exclusively been used for one-way informational messages, which may have educational benefits but does not provide any type of emotional support, which requires some level of interaction and is, for parents of CSHCN, a necessary component of informational support [19,38].

### Mobile Phones and Apps

Apps on mobile phones are some of the newest and most interactive digital modalities for education and communication. On apps, patients are not only getting health information, but also logging their weight, fitness activity, asthma peak flow numbers, insulin levels, food intake, and more, moving patients and families from passive recipients of information to active participants in their health and health care [43]. In addition, apps for mobile phones and tablets are relatively inexpensive, easily customizable to specific patient populations, and widely accessible [44]. Accessibility is growing exponentially, as 68% of Americans owned a smartphone in 2015, up from just over a third in 2011. Smartphone ownership rises with income and education. However, among the 18- to 29-year-olds, smartphone ownership is nearing saturation among all socioeconomic groups [45]. People from low-income households, as well as African American and Latino smartphone users, are more likely to be dependent on their smartphones to obtain information because many have no other access to the Internet [39]. In a study of teenagers and their caregivers in Bronx, New York, USA, most living at well below the federal poverty line and almost entirely African American and Hispanic, 85% had smartphones, and more than 70% reported accessing more than three apps a day. Singh and colleagues suggested that smartphones are becoming more affordable and accessible and, as a result, may allow public health professionals to approach elimination of health disparities in communication [46].

Each study we evaluated that used mobile apps to deliver an intervention focused on providing information and allowing users to record their own information, but lacked any aspect of social support. Any support provided involved feedback from a health care coach or other provider, providing reinforcement but lacking true emotional support. Studies of the effectiveness of apps for health education and support have been mixed. For instance, one study of an app for asthma management showed no significant impact on health outcomes [44], while another showed that app users increased peak flow, increased forced expiratory rate, and reported a high quality of life with fewer unscheduled medical visits [2]. A small study of young women aged 18-30 years in a weight loss program showed a preference for app logs over paper diaries, with 46.2% preferring apps for the program, second only to an online discussion forum. However, retention in the program (67%) was consistent with the low rates of retention and adherence seen in other weight loss programs for young women. Hutchesson and colleagues felt that the women’s interest in technology interventions was promising, however, and noted that health interventions should keep up with new and changing digital platforms [47]. Another study of pregnant women compared patients who used a mobile app to record health information versus women using paper diaries. Patients using the app recorded information more frequently and were more engaged in the intervention than those using paper diaries. They reported the app was easier to use, less of a bother, and more efficient than paper journals, and app users consistently rated their satisfaction with care higher than those using paper diaries [48]. A pilot app developed for parents of high-risk infants allowed parents to record milestones and aspects of care for their baby, record certain aspects of the electronic medical record, and share what they chose with their baby’s care team and on social media. This model of collaborative data collection was not tested but rather was offered as a design model. It offers promising avenues for communication and support for parents of CSHCN, as it allows parents to engage other parents with the app data on their existing digital platforms for support. However, Liu and colleagues also noted that the app’s data collection feature placed a burden on parents who are already overwhelmed to collect and record data about their child, which they may find distressing, as data collection is not typically required of parents of hospitalized infants [2]. Another app for NICU families is available commercially for a fee, but it focuses on support provided to parents via a health coach rather than through peer-to-peer social support [49]. An app called NICU Companion for parents of NICU babies has been developed through the Indiana University School of Medicine [50] as in information-sharing and parent support app. Developers are expanding this platform to allow parents to track baby’s progress, providing information they can then share on social media if they choose. This app is in development and could be a source for further research.

Although evidence is scant, recent studies have suggested that collaborative technology may be more readily accepted by parents than by clinicians [2]. A 2016 study found that physicians and other clinicians were resistant to using patient-collected data from an app unless it didn’t take extra time and was integrated into their normal workflow. Woods and colleagues concluded that clinicians did not yet see the value of patient-collected data and may not be comfortable with the power assigned to the patient as part of his or her own care team. However, they noted that such digital collaboration could work if patients bear most of the responsibility for collecting the data and sharing it with their providers [51]. Apple has introduced its health app for iPhone, which allows individuals to track their own health, fitness, and medical information and, in some cases, share it with their doctors, fitting in neatly with emerging trends in telehealth [52]. Many apps exist or are in development to work with the Apple Health app, creating the option for shared plans of care, patient-provider communication, and other uses that could be helpful to parents of CSHCN. With very few studies focused on the use of apps by parents of CSHCN, understanding the potential and effectiveness of this technology for that population—and its potential for providing social support or enhancing collaborative family-provider partnerships—presents an opportunity for further research.

### Social Media

Social media includes several forums where individuals can share thoughts and experiences with friends and followers by posting on apps and Internet sites such as Facebook, Twitter, Instagram, and other similar sites. Some studies have shown that social media use may help improve health of the users by increasing the perception of having social support and connections, and by creating patient-centered control of what is and isn’t shared. Health improvements have been observed in some social media programs centered on smoking cessation and nutrition [8,16,22,23]. Potential problems with social media may arise if information provided is incorrect, or if a digital divide exists in which vulnerable populations have no access to the Internet and social media [37]. With the rapid advances of social media use and access to mobile phones, however, this digital divide appears to be shrinking. Among all US adults, 65% now use social media sites, up from 7% just 10 years ago. Nearly all (90%) of young adults aged 18-29 years use social media, and use is similar among racial and ethnic groups of all ages. People with higher incomes and higher educational levels of attainment are more likely to use social media, but these differences are far less pronounced among young adults [28]. With the explosion of social media, public health and clinical professionals are increasingly exploring its use for health communication and education [43]. Among the most receptive to social media may be young women, as social media is most commonly used by young adults and women. A 2014 study of how mothers of young children use social media revealed that young moms naturally turn to Facebook during pregnancy and early childhood to share their experiences, just as they have been doing since they were teenagers or college students. In fact, Facebook allows users to make “Expecting a Baby” a Facebook life event, and baby pictures on Facebook are so common, an app called “unbaby.me” allows users to block baby pictures from their Facebook newsfeed [31].

While there is a lack of studies regarding the use of texting and phone apps among parents of CSHCN, social media use is much more commonly studied as an intervention for this population. Social media has been shown to reduce isolation, increase emotional support, and provide useful information, addressing key challenges for parents of young CSHCN. In a 2013 study of parents of children with hydrocephalus [26], parents cited Facebook and YouTube as their preferred sources of information about hydrocephalus and their preferred way to connect with other parents. In that study, 95% of the parents indicated they used social media, and this use cut across racial and socioeconomic lines. A study of a blog and Facebook network of parents of youth with Hirschsprung disease found social media to be a successful way to connect parents of children with rare diseases with support around the world, but noted that this support lacked evidence-based health information [13]. While Facebook is the most popular social media platform, use of social media evolves quickly, requiring ongoing research and consideration of platforms such as Twitter, Instagram, and other emerging platforms of the future.

The support available on social media does more than reduce isolation. Because Facebook groups provide nearly instant access to other parents of CSHCN, parents have the opportunity to learn about systems and services available to their child and how to access them. This informal, online support group helps parents cope with and make sense of the maze of local, state, and national systems of care, as well as the overwhelming amount of information on the Internet and the network of various community services. On social media, parents help other parents navigate this complex web of information and understand what is important to do first, and eventually to be advocates for their child’s needs and rights across health, education, community, and policy arenas [6]. Parents of CSHCN report they have formed meaningful relationships in social media groups, and that they feel less judged by their social media friends than by their friends and family in their “in-person” life. In social media, parents find information and support to address their most pressing needs, which often are things their family and friends don’t understand. These needs include learning ways to help their children’s development, dealing with the emotions of having a CSHCN, handling time demands, finding resources in the community, planning for the future, and understanding their children’s rights. This combination of emotional and informational social support provides parents with important information and real skills that can affect outcomes for their children. In fact, support from other parents on social media has more effect on a parent’s feelings of stress than does their child’s functional level [6]. Other studies offered increasing evidence that online support, particularly through Facebook, improves feelings of well-being, which, consistent with the theory of online social support, assists parents of CSHCN through help with stress management and coping; functional support such as practical advice, information, and assistance; emotional support; and increased perceptions of being supported [16]. These benefits are routinely reported by mothers of CSHCN, as seen in one study focused on moms protesting the closing of an online support network for CSHCN, who praised the empowerment they received from the group, from information shared and emotional support [53]. Parents who receive emotional and informational support from their online friends, especially when those friends are geographically close by or whose children share similar diagnoses, eventually develop the belief they are supported and are able to summon the self-efficacy needed to advocate and pursue services for their CSHCN.

## Discussion

The literature about digital platforms suggests that social media is ideally suited and already in use for supporting and informing parents of CSHCN, but that its primary use is for parent-to-parent support rather than provider-to-parent support. Texting programs have mixed success, with their most significant impact being made in health education and information sharing. The one-way nature of texting programs does not lend itself well to social support, and the multiple factors involved in texting make it difficult to assess. Because of these limitations, texting programs are not ideal for digital communication with parents of CSHCN.

Mobile phone apps are newer formats for health communication, which have shown some success with health outcomes and patient satisfaction. Most are focused strictly on providing information and collecting data, making apps an uncertain platform for social and emotional support. However, the almost total saturation of mobile phone use among young adults, indications that young adults prefer apps to paper-based health programs, barriers to in-person training and support, and the potential relevancy to interactive telehealth and telemedicine make the use of apps an intriguing possibility for parents of young CSHCN. Apps for children with serious health care needs and hospitalizations, such as babies in intensive care units, could allow parents to record important information about their child’s progress, with the potential to use the app as a tool to share updates via social media, thus creating an avenue for social support. Implementation and evaluation of an app-based intervention for parents of CSHCN would be a valuable contribution to what is currently limited literature related to this topic.

For a health care provider such as a hospital, these tools may present a way to encourage parents to connect to support from other parents via social media, without requiring medical providers to directly host a social media site for families. Finding ways to work with social media may become important for pediatric providers because social media seems to offer the most natural and effective means of online social support for parents of CSHCN.

New efforts focusing on digital communication add to the established success of parent-to-parent social support in allowing parents to share information about their child with other parents who have common experiences. Online parent-to-parent support uniquely provides the emotional and informational support that parents of CSHCN often find difficult to obtain in their own families and communities. Nonprofit organizations and, with professional precautions, larger health care organizations can develop interventions around social media strategies, while health care organizations with privacy and practical concerns about maintaining social media interventions can develop mobile phone apps that generate parent-recorded data that families can share with their online social networks, or through telemedicine connections, with their child’s doctor. Apps that allow parents to easily share their child’s progress with social networks could be a focus of future app development and research. Location-based, opt-in apps could even serve to connect parents with children who have similar diagnoses in given locations, such as in hospital cafeterias. Partnerships with community family-to-family organizations who already offer online support to families of CSHCN could be another option for health care providers, providing an avenue to reach families with information through their community partners’ social media channels. In these ways, parents learn about their children and their needs from health care professionals, share this information with their social networks, and then receive from those networks the emotional and informational support that applies to real life as a parent of a CSHCN. Exploration of potential online programs to enhance provider-parent relationships should include research into physicians’ and other medical professionals’ goals and barriers around online family support. Additionally, online support among youth with special health care needs—connecting them with other young people with similar diagnoses—would be another new area of research within the framework of online social support. In addition, efforts to further establish associations between social support and positive health outcomes can lead to greater acceptance of such programs and better options for funding.

Online social support has been shown to be effective in helping parents accept their child’s diagnosis and develop the skills they need to help their child in the days and years to come. Additional research-based evidence about online tools for communication and partnership between parents of CSHCN and physicians could advance the adoption of such tools by health care providers if such tools are shown to be effective. Digital interventions that work to inform, educate, and empower parents of CSHCN, including interventions that work within the framework of online social support, should be the focus of future research, with the goal of improving outcomes for CSHCN and their families.

## Abbreviations

ADHD: attention-deficit/hyperactivity disorder
CSHCN: children with special health care needs
ERIC: Education Resources Information Center
NICU: neonatal intensive care unit
SMS: short message service
